# Supplementary material for: Deletion of Slc1a4 Suppresses Single Mauthner Cell Axon Regeneration In Vivo through Growth-Associated Protein 43
Source: Int J Mol Sci. 2024 Oct 11;25(20):10950. doi: 10.3390/ijms252010950 (PMC11507230; doi:10.3390/ijms252010950)
Supplement: Supplementary file 1 [file ijms-25-10950-s001.zip › captions for Figures S1–S3.docx]

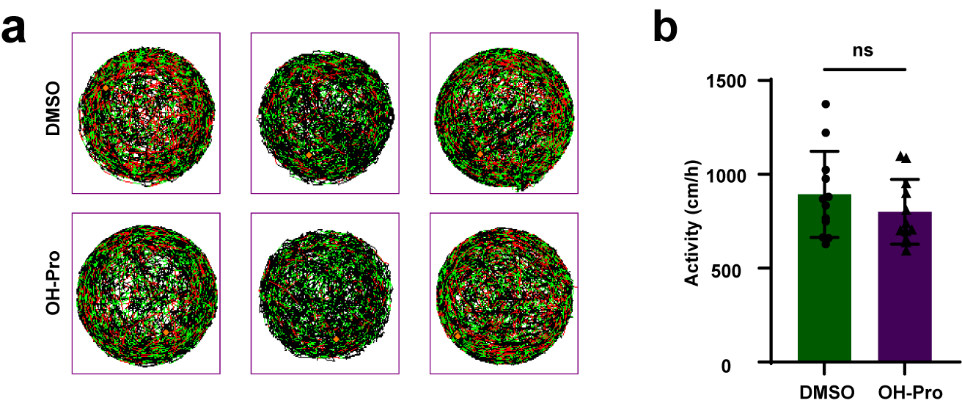

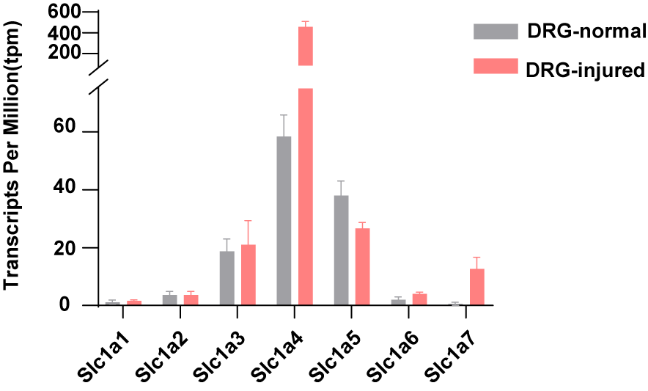
**Figure S1.** Expression levels of amino acid transporter family 1 before and after DRG injury.


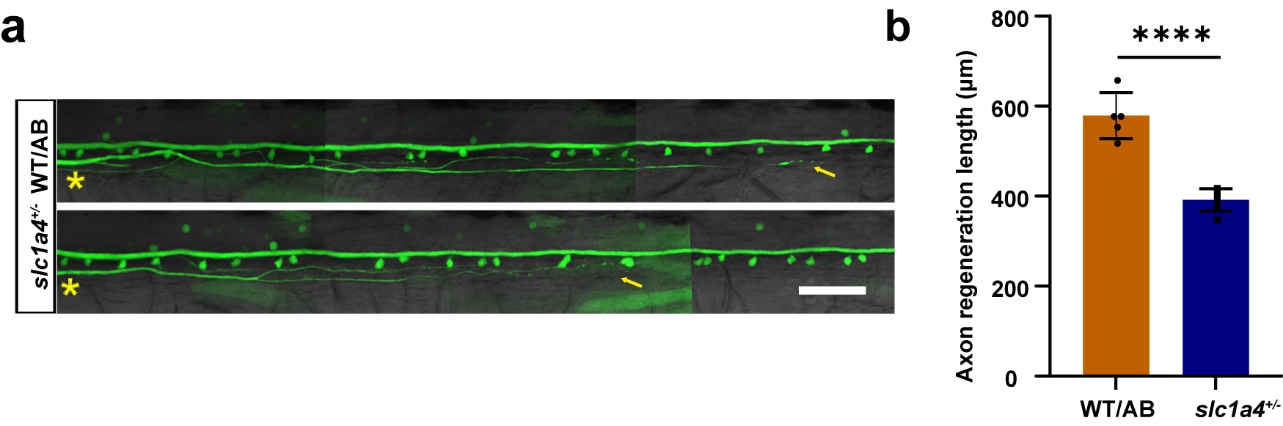
**Figure S2.** Spontaneous locomotor behavior in inhibitor-treated zebrafish. (**a, b**) The line illustrates the 6 dpf zebrafish larvae’s swimming trajectory differences from the DMSO and OH-Pro groups evaluated over 1 h Data shown as mean ± sem. Assessed by unpaired *t*-test. ns, not significant.

**Figure S3.** Axonal regeneration in heterozygotes of the *slc1a4* mutant line. (**a, b**) Representative diagram of confocal imaging of M-cells axon regeneration between WT and *slc1a4*^+/-^. Data shown as mean ± sem. WT/AB: 579.2 ± 22.99 μm, *slc1a4*^+/-^: 391.7 ± 10.14 μm. scale bar,50 μm. Assessed by unpaired *t*-test. **** *p* < 0.0001.
