# Supplementary figures and images for: Deletion of Slc1a4 Suppresses Single Mauthner Cell Axon Regeneration In Vivo through Growth-Associated Protein 43
Source: Int J Mol Sci. 2024 Oct 11;25(20):10950. doi: 10.3390/ijms252010950 (PMC11507230; doi:10.3390/ijms252010950)

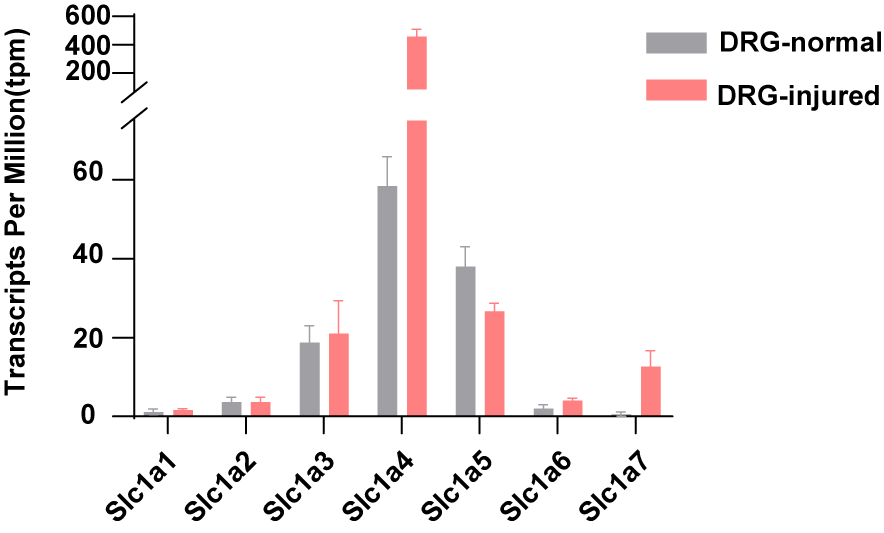

Supplement: Supplementary file 1 [file ijms-25-10950-s001.zip › Figure S1.tif]

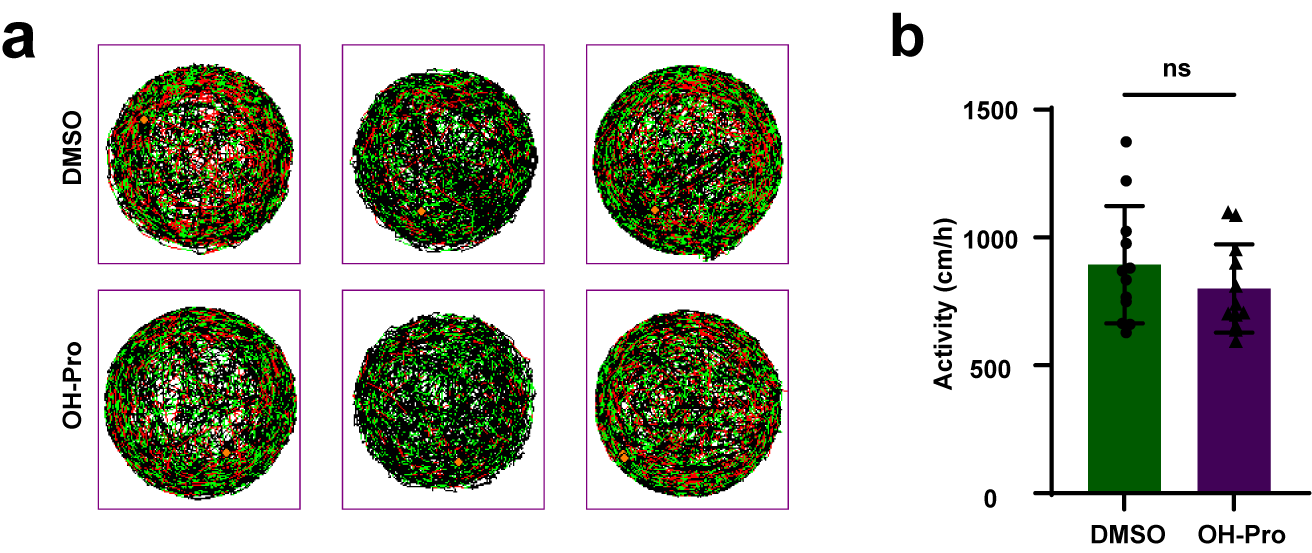

Supplement: Supplementary file 1 [file ijms-25-10950-s001.zip › Figure S2.tif]

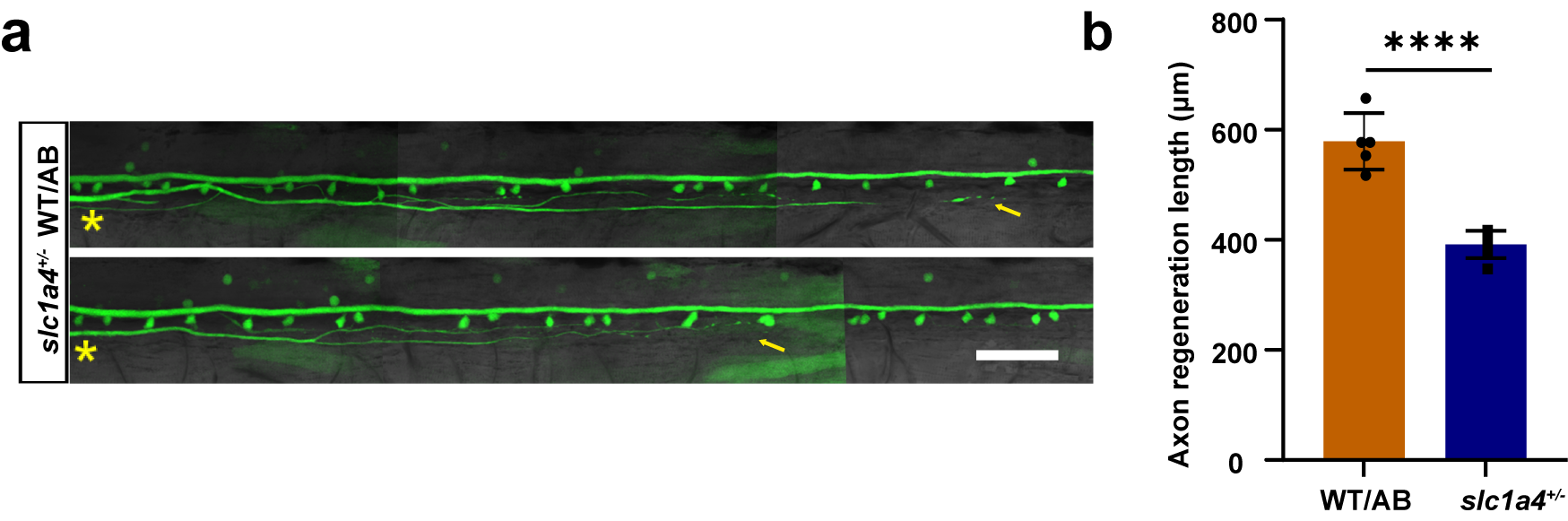

Supplement: Supplementary file 1 [file ijms-25-10950-s001.zip › Figure S3.tif]
